# Supplementary material for: Invasion and migration of spatially self‐limiting gene drives: A comparative analysis
Source: Evol Appl. 2018 Jan 4;11(5):794–808. doi: 10.1111/eva.12583 (PMC5978947; doi:10.1111/eva.12583)

Appendix 1: Alternative payload dominance patterns

In addition to the multiplicative costs of payload presented in the main text, we also analyze the performance of the three gene drives with dominant, recessive and additive payload costs. To incorporate these dominance patterns, we modify the component of genotypic fitness attributed to the effects of the payload genes. Such that individuals heterozygous for the payload allele pay a fitness cost of$\delta s_{p}$, i.e. have a relative fitness component $\left( 1-\delta s_{p} \right)$, where $\delta$ has a value of 1, 0 or ½ for dominant, recessive and additive costs of the payload respectively. Individuals homozygous for the payload allele pay the full cost $s_{p}$ with a relative fitness component $\left( 1-s_{p} \right)$, while wild-type homozygotes have a relative fitness component of 1. Note, that in the case of the one-locus underdominance gene drive, heterozygotes are not viable. Therefore, changing the payload cost patterns does not change the results for one-locus underdominance.

Overall, changing the payload dominance pattern affects the results only quantitatively. That is, the relative level of localization of different gene drives with respect to each other is not influenced by the dominance pattern (Figures S1, S2, S3). Certain dominance patterns do change the ease with which the gene drives can spread in a population.

***A) Performance in isolated populations***

Changing the dominance pattern for the payload gene does not have a severe effect on the conditions under which each of the gene drives can spread successfully in an isolated population (Figure S1). One potentially important change is that when the payload costs are dominant, the equilibrium payload frequency for a two-locus underdominance drive is close to fixation when it successfully spreads the gene drive. This is in contrast with other dominance patterns (see Figure 3B in the main text, or figures S2 & S3 below), where the equilibrium frequency for the two-locus underdominance drive is much lower than 1. This is because the wild-type alleles cannot be maintained in the population in heterozygotes, as heterozygotes suffer the full costs of the payload.

**Figure S1: Dominant payload cost - Contour plots show the mean frequency of the payload allele in the 100 generations following a single introduction of modified organisms. The yellow dot on each contour plot shows the same conditions as in Figure 3 in the main text, and are included for easier visual comparison.**

**
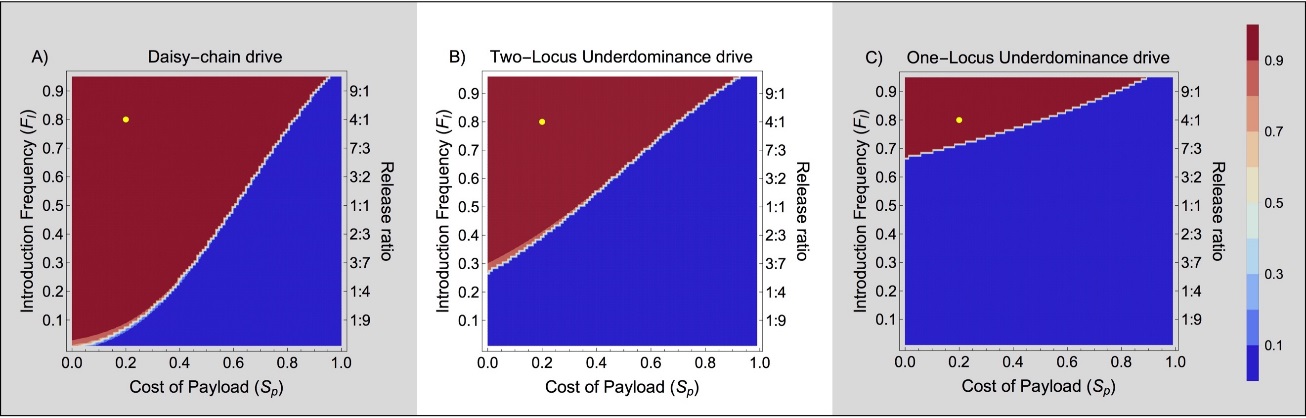
**

**Figure S2: Recessive payload cost Contour plots show the mean frequency of the payload allele in the 100 generations following a single introduction of modified organisms. The yellow dot on each contour plot shows the same conditions as in Figure 3 in the main text, and are included for easier visual comparison.**


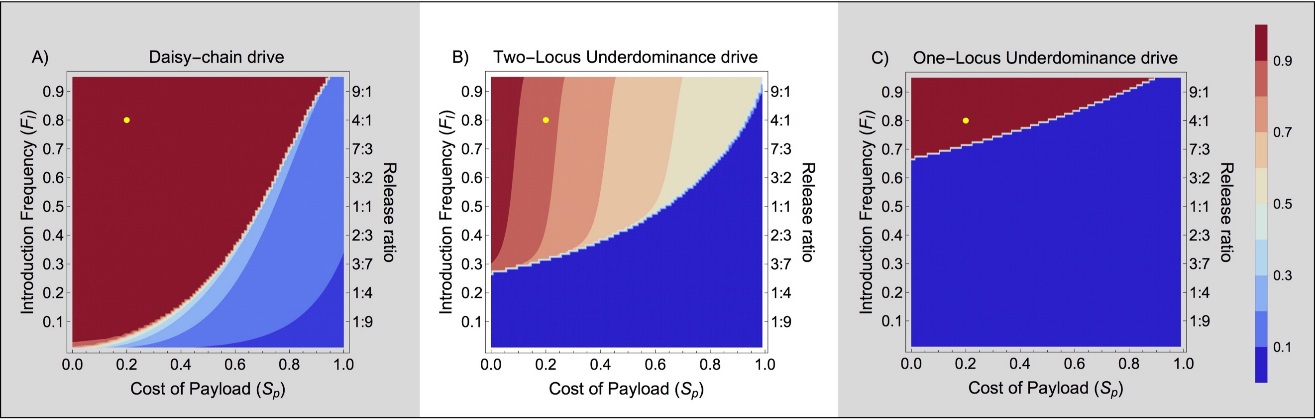


**Figure S3: Additive payload cost Contour plots show the mean frequency of the payload allele in the 100 generations following a single introduction of modified organisms. The yellow dot on each contour plot shows the same conditions as in Figure 3 in the main text, and are included for easier visual comparison.**

**
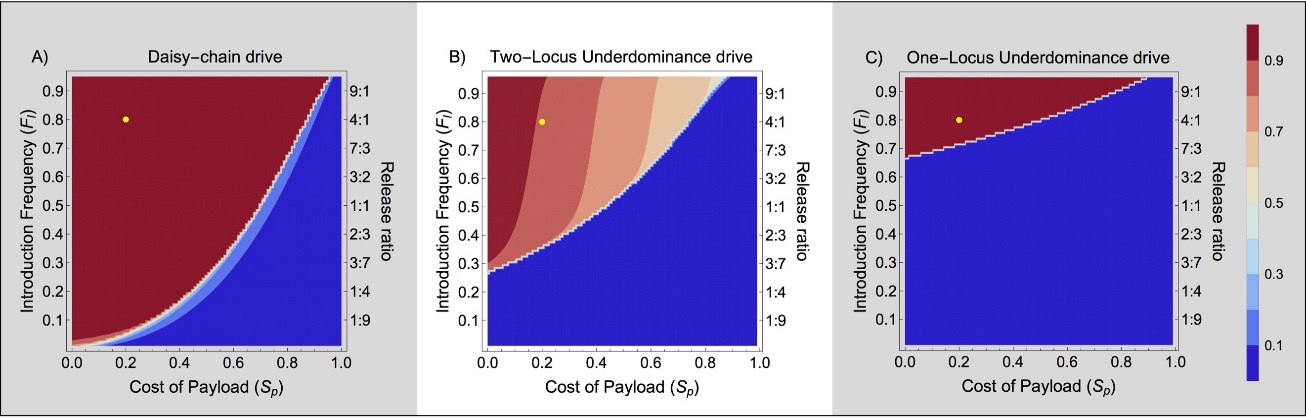
**

***B) Level of Localization***

The different payload dominance patterns do not change the relative localization level of the different gene drives with respect to each other. The one-locus underdominance drive (which is not affected by changes in dominance patterns) is most difficult to spread successfully in the target population, but when it does spread, it remains localized. The two-locus underdominance drive is relatively easier to spread and relatively less localized, while the daisy chain drive is the easiest to spread, and is also the least localized, when comparing within a particular dominance pattern.

When comparing different payload dominance patterns within a gene drive, the daisy chain drive and the two-locus underdominance drive can be slightly more localized when they carry a dominant payload gene than a payload gene with a recessive, additive or multiplicative payload (compare Figures S4, S5, S6 and Figures 5 in the main text). As mentioned before, the one-locus underdominance drive is not affected by different payload dominance patterns, because only homozygotes are viable.

**Figure S4: Dominant payload costs - Contour plots show the mean frequency of the payload allele in the first 100 generations in the target and the neighboring population. The yellow dots have the same coordinates as those in the corresponding panels in Figure 5 in the main text (which have multiplicative payload costs), and are shown here to aid visual comparison.**

**
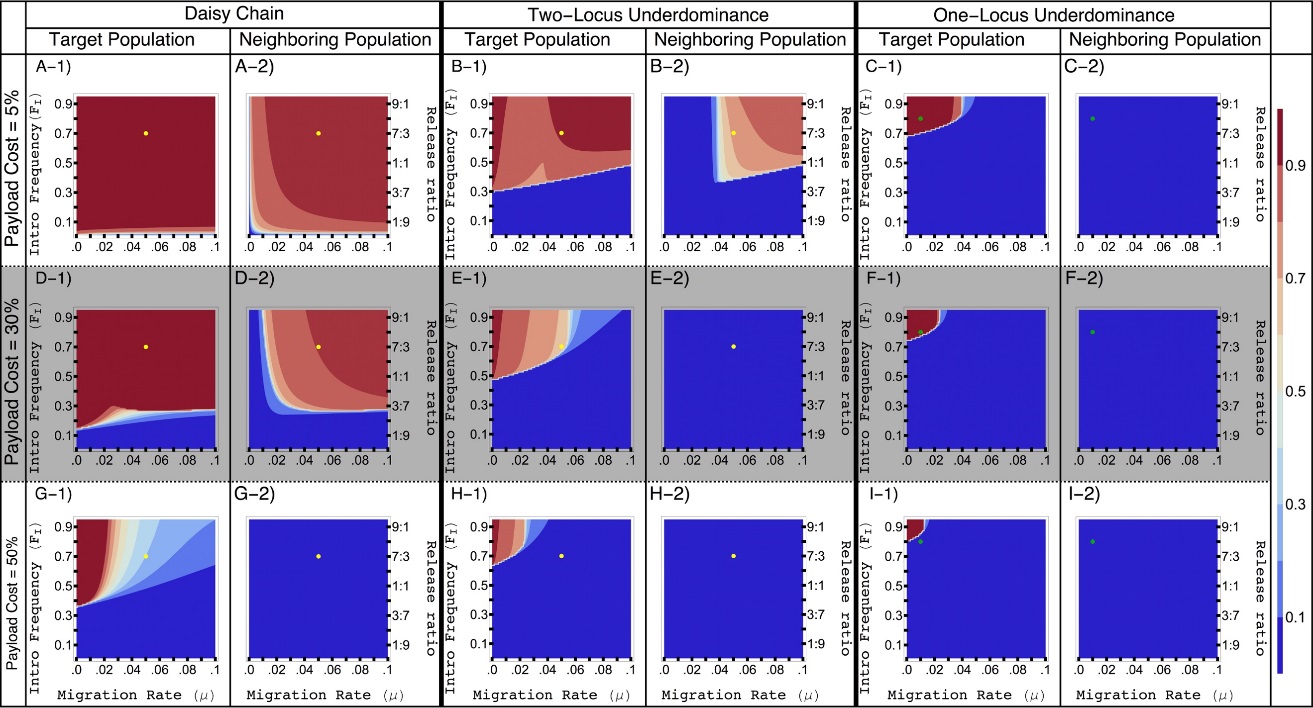
**

**Figure S5: Recessive payload costs - Contour plots show the mean frequency of the payload allele in the first 100 generations in the target and the neighboring population. The yellow dots have the same coordinates as those in the corresponding panels in Figure 5 in the main text (which have multiplicative payload costs), and are shown here to aid visual comparison.**

**
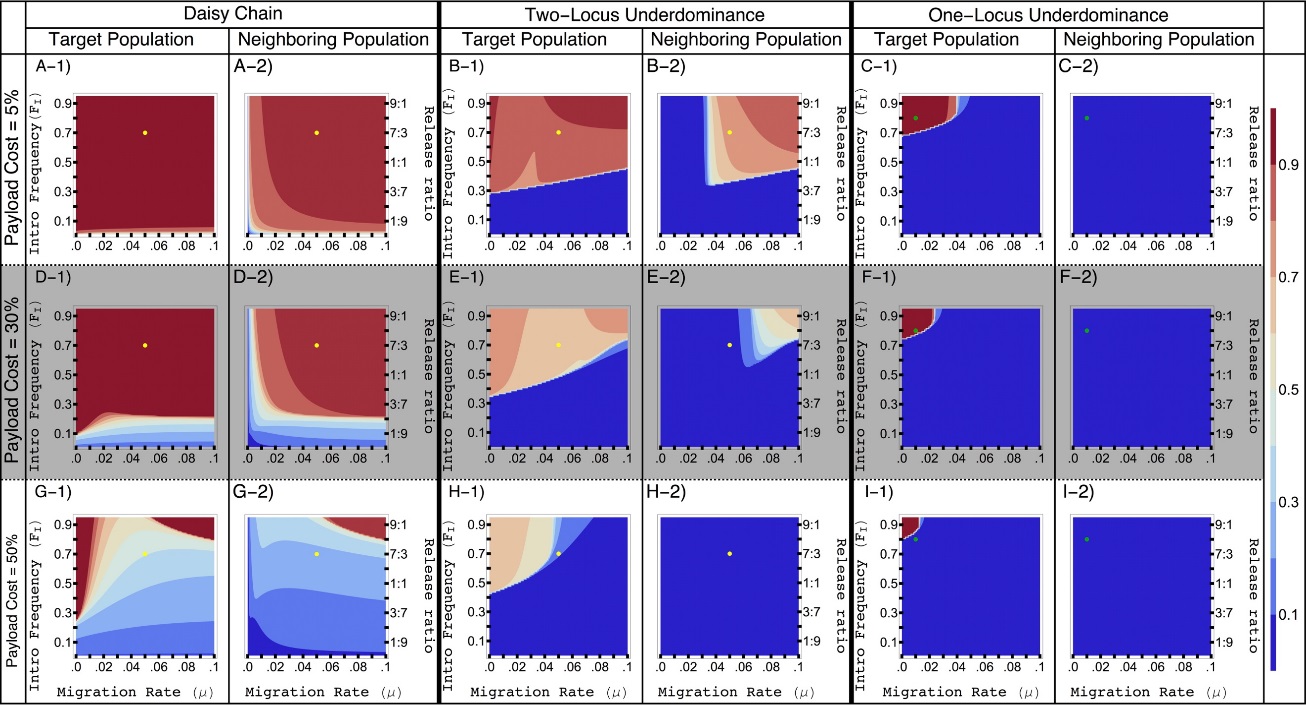
**

**Figure S6: Additive payload costs - Contour plots show the mean frequency of the payload allele in the first 100 generations in the target and the neighboring population. The yellow dots have the same coordinates as those in the corresponding panels in Figure 5 in the main text (which have multiplicative payload costs), and are shown here to aid visual comparison.**

**
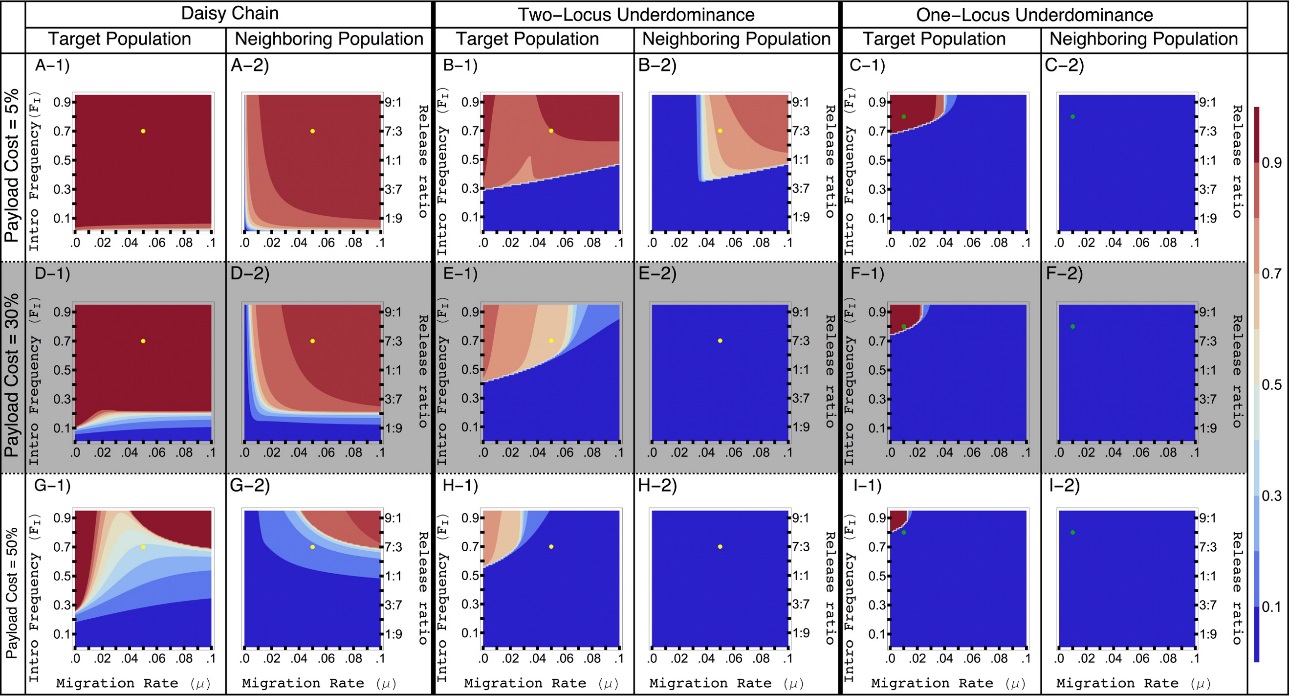
**

Appendix 2: Variations of the Daisy-chain drive

Of the three gene drives addressed in this study, the daisy chain drive is perhaps the most malleable; the number of elements in the daisy chain can be altered, with each additional element resulting in a stronger driving effect (Noble et al. 2016). It is not clear how feasible it would be to construct a daisy chain drive with a large number of elements. Indeed, we expect that a daisy chain drive with more than three elements would be less localized than the three-element drive discussed in the main text. But in addition to changing the number of elements in the drive, the fitness effects of the additional elements (besides the payload gene) may be modifiable as well (Noble et al. 2016). The basic structure of the daisy chain drive (Noble et al. 2016), as addressed in the main text, has non-payload elements without any independent fitness costs (they do not alter fitness other than through the drive mechanism). Noble et al. (2016) discuss a version of the daisy chain drive where the non-payload elements bear a fitness cost. We performed our analysis with a 10% independent fitness cost for each of the non-payload drive elements (elements B and C in a three-element daisy chain drive).

We find that adding a fixed fitness cost to each of the non-payload elements has an effect very similar to that of increasing the cost of only the payload element (compare Figure 5 and Figure S7). Moreover, even if only one of the non-payload element has a very high non-homing-related fitness cost (50%), the efficiency of the daisy chain drive is greatly reduced (Costly second element (element B): Figure S8; Costly third element (element C): Figure S9).

**Figure S7: Localization analysis of Daisy-chain drive where the two non-payload elements (B and C) each carry a 10% multiplicative fitness cost, in addition to the costs associated with inefficient homing. Contour plots show mean frequency of the payload allele in the first 100 generations in the two populations, after a single introduction of modified organisms into the target population. The yellow dots on each contour plot have the same coordinates (Introduction frequency =70%, μ = 0.05) as the dots on the corresponding figure (Figure 5 A, D & G) in the main text, and are shown here for easier visual comparison. Payload costs shown for each row are the costs incurred by individuals homozygous for the payload transgene.**


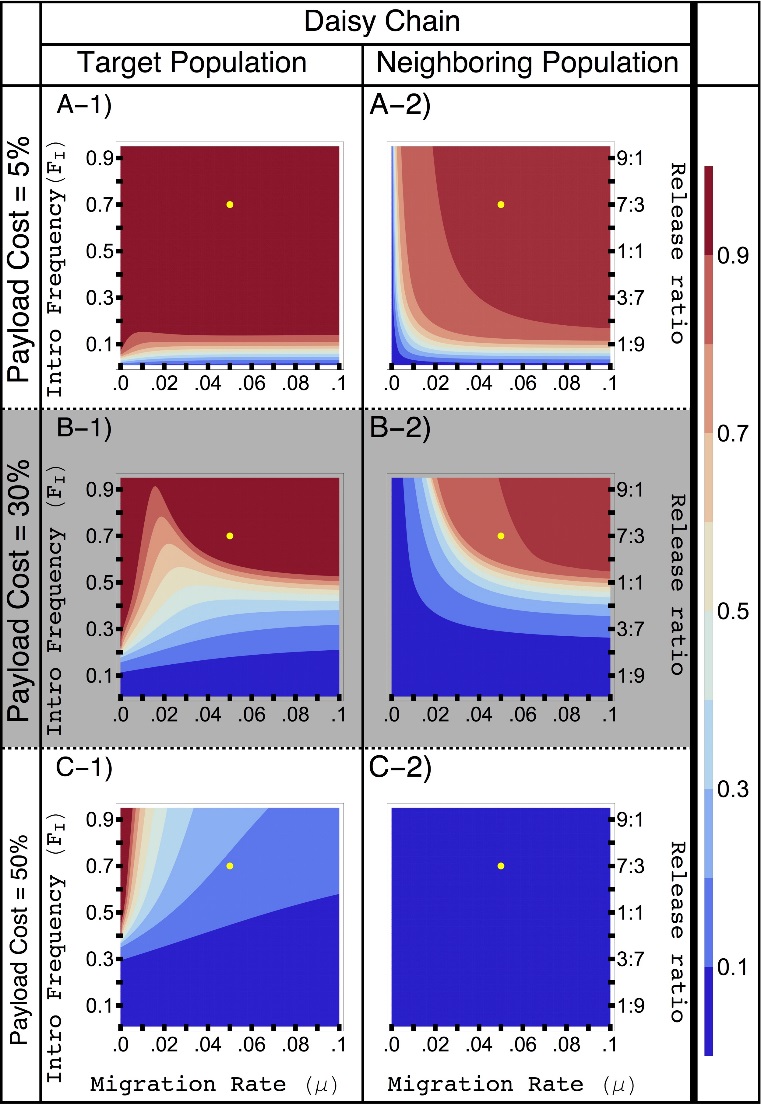


**Figure S8: Localization analysis of a Daisy-chain drive with an element B that carries a 50% multiplicative, non-homing-related fitness cost. Contour plots show mean frequency of the payload allele in the first 100 generations in the two populations, after a single introduction of modified organisms into the target population. The yellow dots on each contour plot have the same coordinates (Introduction frequency =70%, μ = 0.05) as the dots on the corresponding figure (Figure 5 A, D & G) in the main text, and are shown here for easier visual comparison. Payload costs shown for each row are the costs incurred by individuals homozygous for the payload transgene. Element C here does not confer fitness costs besides those associated with inefficient homing.**


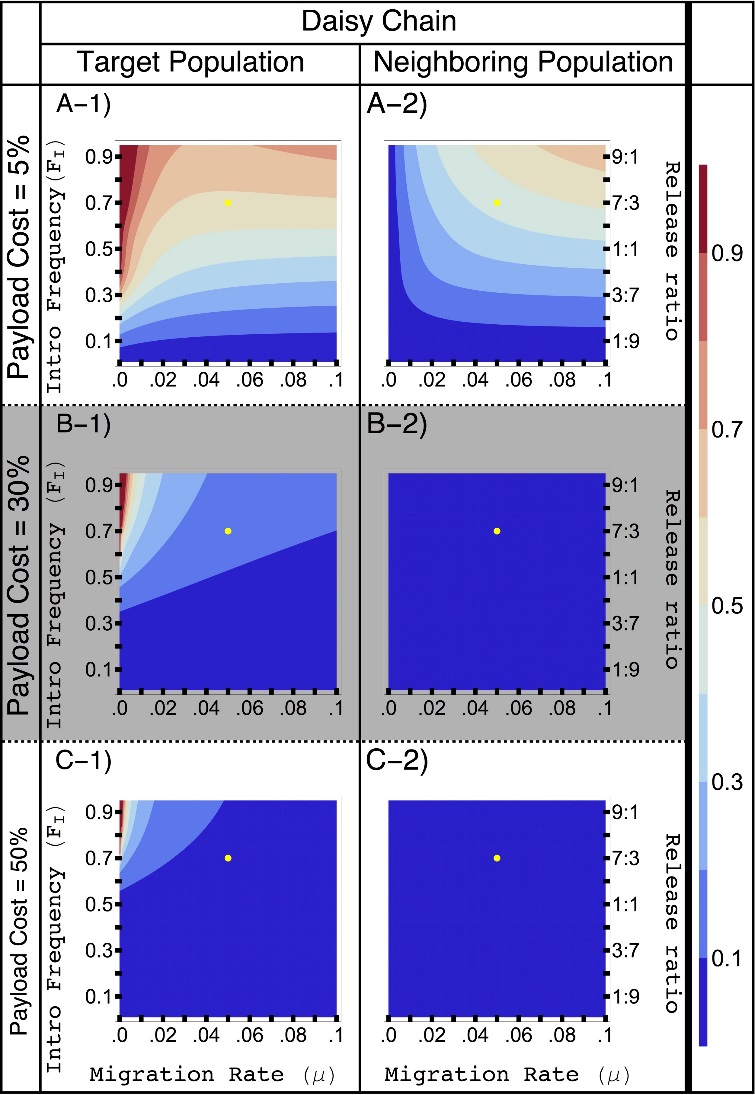


**Figure S9: Localization analysis of a Daisy-chain drive with a element C that carries a 50% multiplicative, non-homing-related fitness cost. Contour plots show mean frequency of the payload allele in the first 100 generations in the two populations, after a single introduction of modified organisms into the target population. The yellow dots on each contour plot have the same coordinates (Introduction frequency =70%, μ = 0.05) as the dots on the corresponding figure (Figure 5 A, D & G) in the main text, and are shown here for easier visual comparison. Payload costs shown for each row are the costs incurred by individuals homozygous for the payload transgene. Element B here does not confer fitness costs besides those associated with inefficient homing.**


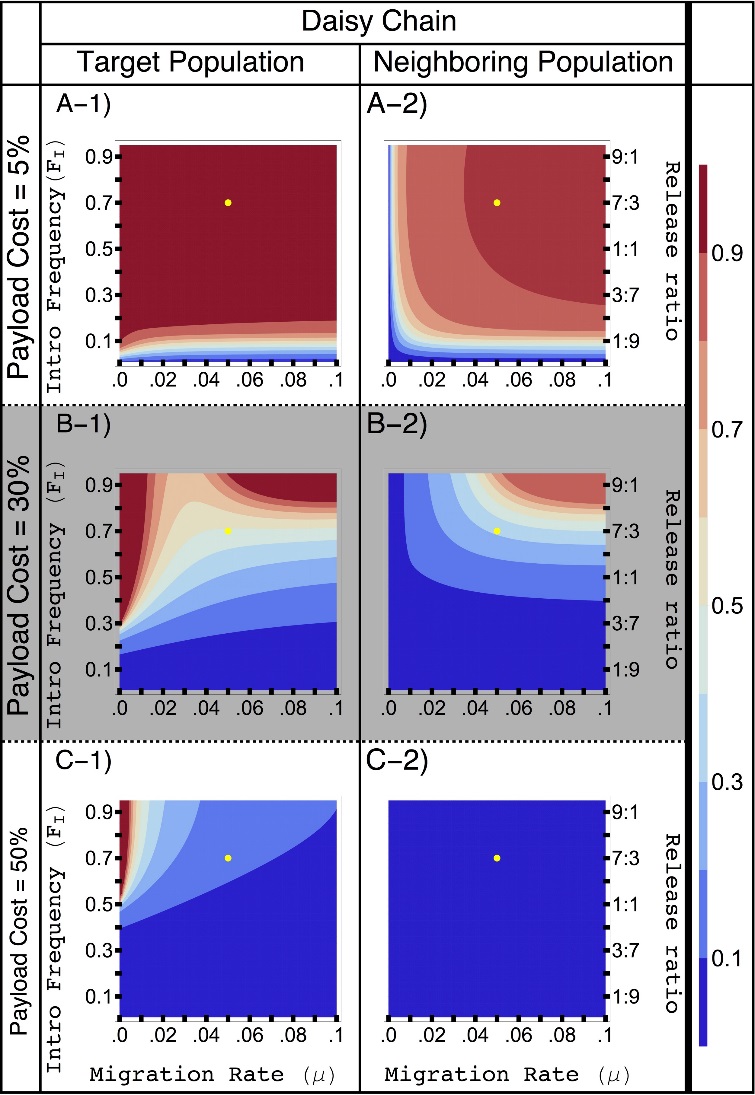


Appendix 3: Migration independent of population size

In the main text, we describe analyses for bidirectional migration rates that account for differences in the sizes of the two adult population. We also analyzed the performance of gene drives with migration rates that are fixed, and do not change with the relative population size. Biologically, this is analogous to assuming that the number of adults in the target and the neighboring populations remain equal irrespective of differences in mortality during earlier stages that may arise as the gene drive spreads.

The results shown below do not qualitatively differ from the ones shown in the main text about which gene drives are more localized. For each gene drive, having fixed migration rates does slightly reduce localization levels (compare Figure 5 with Figure S10). It also becomes relatively easier to spread each gene drive in the target population. This follows intuition, because unlike the case shown in the main text, here migration out of the target population does not decrease due to gene drive-related mortality in earlier life stages, and the effective immigration into the target population does not increase either.

**Figure S10: Contour plots show mean frequency of the payload allele in the first 100 generations in the target and the neighboring population for the three gene drives, with three different homozygous multiplicative costs of payload each. Migration rates do not change with relative population size. The yellow and green dots have the same coordinates as those in the corresponding panels in Figure 5 in the main text, and are shown here to aid visual comparison.**


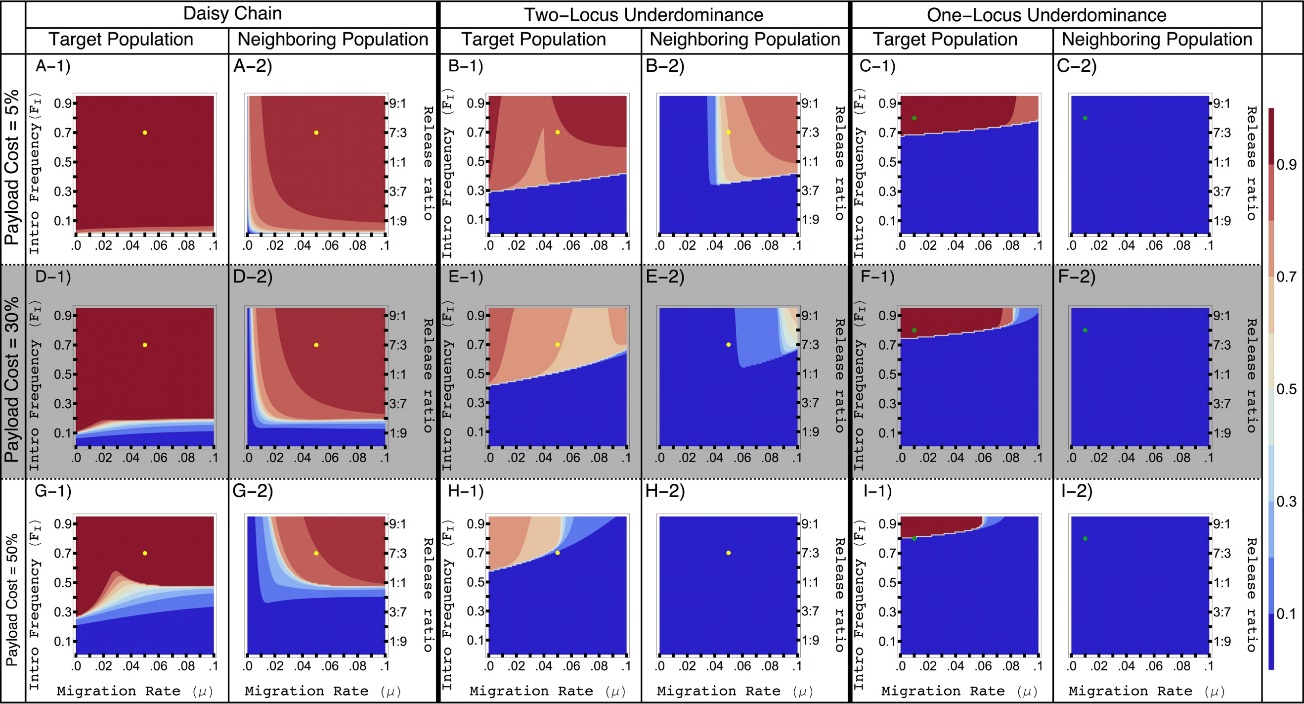


Appendix 4: Capacity for population suppression with *recessive*, female-limited payload expression

In addition to the case of multiplicative female-limited expression of payload cost shown in the main text, we also compared the genetic load that can be generates using a payload gene with a recessive female-limited expression. The capacity for population suppression of a daisy-chain drive with a recessive female-limited payload expression is only slightly lower than that of a daisy-chain drive based on a multiplicative female-limited payload expression (Figure S11A). This is because in either case the daisy-chain drive can push the payload to very high frequencies very quickly, leaving few individuals heterozygous for the payload.

In case of the two-locus underdominance drive, a recessive female-limited payload expression severely reduces the genetic load that can be achieved by the drive (Figure S11B). The two-locus underdominance drive does not push the payload to fixation, leaving a large number of individuals that are heterozygous for the payload. With a recessive payload expression, this limits the total genetic load in each generation.

Again, note that in the case of the one-locus underdominance drive, only transgenic heterozygotes (and wild-type homozygotes) are viable. Therefore, the payload is expressed only in homozygous state, and the dominance pattern does not affect the results (Figure S11C).

**Figure S11: Contour plots show mean of genetic load on an isolated population within the first 20 generations after a single introduction of gene drives with recessive female-limited payload cost.**


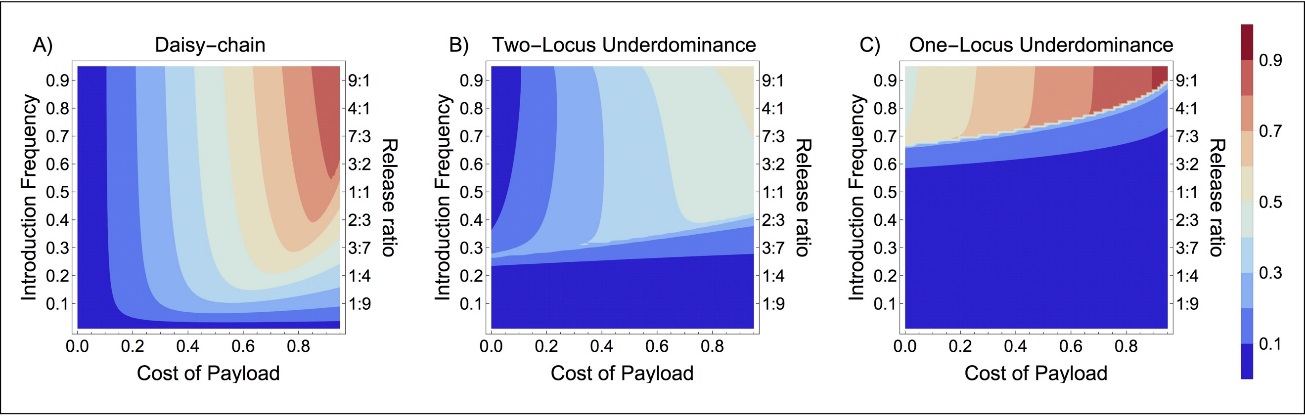

Supplement: Supplementary file 1 [file EVA-11-794-s001.docx]
